# The jasmonate receptor COI1 is required for AtPep1-induced immune responses in *Arabidopsis thaliana*

**DOI:** 10.1186/s13104-018-3628-7

**Published:** 2018-08-03

**Authors:** Danalyn R. Holmes, Lauren E. Grubb, Jacqueline Monaghan

**Affiliations:** Biology Department, Queen’s University, 116 Barrie St, Kingston, ON K7L 3N6 Canada

**Keywords:** Immunity, Jasmonate, COI1, AtPep1, DAMP

## Abstract

**Objective:**

Plant cells detect the presence of potentially pathogenic microorganisms in the apoplast via plasma membrane-localized receptors. Activated receptors trigger phosphorylation-mediated signaling cascades that protect the cell from infection. It is thought that signaling triggered by the detection of exogenous signals, such as bacterial flagellin, can be amplified by endogenous signals, such as hormones or debris caused by cell damage, to potentiate robust immune responses. For example, perception of flagellin and other microbial molecules results in increased expression of endogenous *PROPEP* transcripts that give rise to AtPep peptides which also activate immune signaling. Phytohormones such as methyl-jasmonate also induce *PROPEP* expression, suggestive of additional hormone-mediated feedback loops that similarly amplify immune signaling. The current study aimed to determine if perception of jasmonate is genetically required for AtPep1-induced immune responses in *Arabidopsis thaliana.*

**Results:**

We assessed several AtPep1-induced immune responses in plants expressing a non-functional variant of the jasmonate receptor CORONATINE-INSENSITIVE 1 (COI1). We found that *coi1*-*16* mutants are severely compromised in some AtPep1-induced immune responses, while other AtPep1-induced responses are maintained but reduced. Our findings build on previously published work and suggest that JA perception plays a role in immune responses triggered by AtPep1.

**Electronic supplementary material:**

The online version of this article (10.1186/s13104-018-3628-7) contains supplementary material, which is available to authorized users.

## Introduction

Plants lack a humoral immune system and rely solely on the innate ability of each cell to detect potentially harmful pathogens and defend against disease. Plasma membrane-localized pattern recognition receptors (PRRs) bind ‘non-self’ molecules characteristic of entire classes of microbes known as pathogen-associated molecular patterns (PAMPs), which are typically integral to microbial lifestyles and are thus under strong selection pressure [1]. Examples include bacterial proteins flagellin and Elongation Factor Tu (EF-Tu), which are recognized in *Arabidopsis thaliana* by receptor kinases FLAGELLIN SENSING 2 (FLS2) and EF-Tu RECEPTOR (EFR), respectively [2–4]. PRRs also bind ‘infectious-self’ molecules known as damage-associated molecular patterns (DAMPs), such as cell wall fragments or small peptides that are thought to be released by the plant cell during pathogen invasion and/or wounding [1, 5]. For example, the Arabidopsis PRRs AtPEP RECEPTOR KINASE 1 (PEPR1) and PEPR2 bind endogenous AtPep peptides resulting in the activation of immune responses [6–9]. Many PRRs function in protein complexes, requiring regulatory co-receptors for full activation and subsequent signal transduction [1, 10]. Upon ligand binding, FLS2, EFR, and PEPR1/2 each form a complex with the receptor-like kinase BRI1-ASSOCIATED RECEPTOR KINASE 1 (BAK1) [11–16]. PRR activation and complex formation lead to pattern-triggered immunity (PTI), characterized by an influx of Ca^2+^, the activation of receptor-like cytoplasmic kinases (RLCKs), a rapid and transient apoplastic oxidative burst, the activation of mitogen-activated and calcium-dependent protein kinases (MAPK and CDPKs), and transcriptional reprogramming resulting in a basal immune response that is effective against most potential pathogens [1, 17].

Interplay between plant immune and hormone signaling has been observed in several systems [18]. In particular, the antagonistic roles of salicylate (SA) and jasmonate (JA) in defense against biotrophic and necrotrophic pathogens has been well documented [19], and the involvement of these and other phytohormones in pattern-triggered signaling has also been observed [20]. As one example, perception of several AtPeps causes an increase in the classical SA- and JA-triggered marker genes *PATHOGENESIS RELATED*-*1* (*PR*-*1*) and *PLANT DEFENSIN 1.2* (*PDF1.2*), and expression of AtPep precursor *PROPEP* genes is induced by treatment with methyl-salicylate (MeSA), methyl-jasmonate (MeJA), immunogenic peptides, as well as pathogen infection and herbivore feeding [7, 8, 21]. These and other observations [22, 23] suggest a feedback loop that amplifies immune signaling following pathogen infection. Here we present data demonstrating that the JA receptor CORONATINE-INSENSITIVE 1 (COI1) is genetically required for AtPep1-induced immune outputs to varying levels. Our work builds on earlier observations [24] and supports a role for JA signaling in AtPep1-induced responses.

## Main text

### Methods

*Arabidopsis thaliana* ecotype Columbia (Col-0) and previously described mutants *bak1*-*5* [25]*, glabra1* (Col *gl1*) [26]*, SA*-*induction deficient 2*-*2* (*sid2*-*2*) [27], and *coi1*-*16* (in the Col *gl1* background) [28] were used in this study. These lines have been propagated in lab environments and were not collected from the wild; see Acknowledgements section for the source of each seed line. For sterile assays, seeds were surface-sterilized and sown on half-strength Murashige & Skoog (MS) agar plates (0.8%) and stratified in the dark at 4 °C for 3 days before being exposed to a 12 h photoperiod. For soil assays, seeds were similarly stratified and seedlings were grown on soil in controlled environment chambers at 22 °C with 30% humidity in a 10 h photoperiod. Immunogenic elicitor peptides flg22, elf18, and AtPep1 were synthesized by EZ Biolabs (USA) and used in seedling growth inhibition, oxidative burst, and MAPK activation assays as described previously [29]. For gene expression assays, RNA was extracted from twelve 2-week-old seedlings grown in sterile liquid culture using the Aurum Total RNA Mini Kit (BioRad) and mRNA was reverse transcribed using an oligo dT_18_ primer and SuperScript III (Invitrogen) following the manufacturer’s directions. Quantitative real-time PCR was performed using SsoAdvanced Universal SYBR Green Supermix (BioRad) and measured on a CFX96 Touch Real-Time PCR Detection System (BioRad). Melting curve analysis confirmed that all primer pairs amplify a single product; primer sequences are listed in Additional file 1.

## Results and discussion

**Fig. 1:**
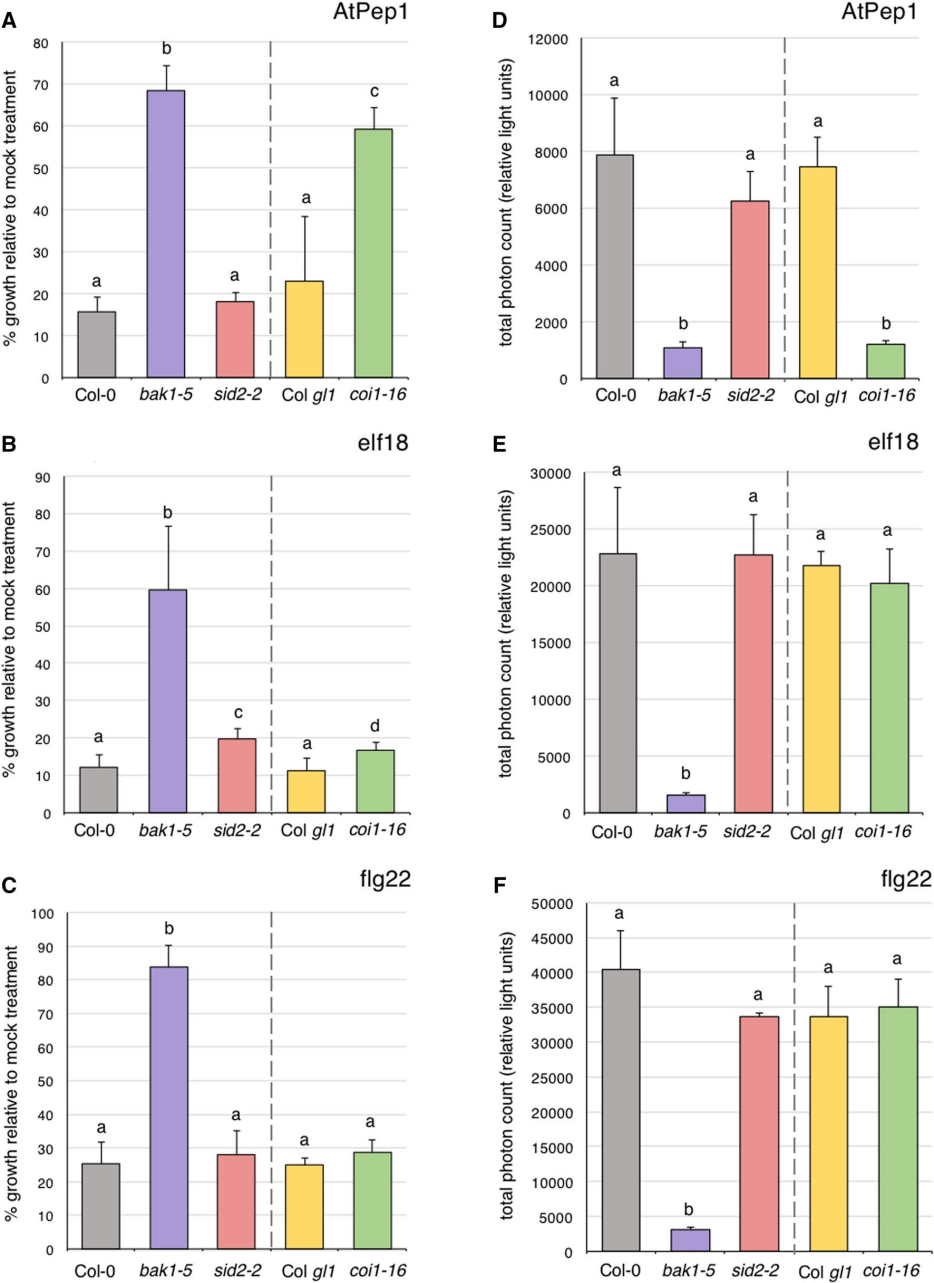
AtPep1-induced seedling growth inhibition and oxidative burst in *coi1*-*16* mutants. **A**–**C** Seedling inhibition after 10 days of continual growth in sterile liquid MS media containing 500 nM AtPep1 (**A**), 100 nM elf18 (**B**), or 100 nM flg22 (**C**) in the indicated genotypes. Values are  % means of seedling fresh weight + standard deviation (*n *= 6 seedlings), relative to average fresh weight in MS media alone. **D**–**F** Oxidative burst on 5-week-old soil-grown plants following treatment with 500 nM AtPep1 (**D**), 100 nM elf18 (**E**), or 100 nM flg22 (**F**) in the indicated genotypes. Relative light units were recorded using the LUM module on a SpectraMax Paradigm plate reader for 40 min at 2 min intervals using an integration time of 1000 ms. Values are means + standard deviation (*n *= 6 plants). Experiments were performed independently on three sets of plants with similar results; a single representative experiment is shown for each assay. Statistically significant groups (*p* < 0.05) are indicated with lower-case letters based on a one-way ANOVA followed by Tukey’s post-test

*Arabidopsis* seedlings constantly exposed to immunogenic peptides display severe growth inhibition, presumably due to continual activation of immune signaling that diverts resources away from normal growth and development. Although cross-talk between immune and hormone pathways has been well-demonstrated [18, 19], how plant hormone signaling influences immune-induced growth inhibition is largely unknown. While performing experiments for other projects in our lab, we found that the JA receptor mutant *coi1*-*16* [28] was almost as insensitive as the immunodeficient mutant *bak1*-*5* [25] to AtPep1-induced seedling inhibition (Fig. 1A). We found this to be specific to AtPep1, as sensitivity to the EF-Tu epitope elf18 and the flagellin epitope flg22 was comparable to controls (Fig. 1B, C). Comparatively, the mutant *sid2*-*2*, which cannot synthesize SA due to lack of functional isochorismate synthase [27], was not affected in these assays (Fig. 1A–C). To account for any inherent growth differences between genotypes, total fresh weight of seedlings grown in the presence of immunogenic peptides was calculated relative to their growth in MS media. All genotypes used in this study grew similarly in MS media as shown in Additional file 2.

As seedling inhibition is considered a late immune response, we extended our analysis to test if JA perception via COI1 is also required for an earlier immune response such as the RESPIRATORY BURST OXIDASE HOMOLOG D (RBOHD)-mediated burst of reactive oxygen species (ROS). We found that while elf18- and flg22-induced ROS was unaffected in *coi1*-*16* compared to controls, AtPep1-induced ROS was as severely inhibited as in *bak1*-*5* mutants (Fig. 1D–F), indicating that JA perception is required quite early in AtPep1-triggered signaling. It was previously shown that *coi1*-*1* mutants are compromised in AtPep1-induced seedling growth inhibition, ROS, and ethylene production, while flg22-triggered responses were not affected [24]. Thus, our study using the independent *coi1*-*16* allele, which is in the Col *gl1* background [28], corroborates previous work. The same phenomenon was observed in the *allene oxide synthase* (*aos*) mutant which cannot synthesize JA [24], suggesting that both JA biosynthesis and perception are genetically required for AtPep1-mediated immune signaling.

While some PTI responses are directly linked via phosphorylation cascades, genetic evidence supports parallel activation of other outputs downstream of PRR activation [1]. For example, elicitor-induced MAPK activation and RBOHD-dependent ROS are rapid and transient responses that occur simultaneously, both peaking at around 10 min following PRR activation [30]. While activation of RBOHD has been directly linked to phosphorylation by CDPKs and RLCKs [31–34], evidence from several studies [35–37] has suggested that the NADPH oxidase RBOHD and MAPKs are independently activated. For example, flg22-induced activation of MPK6, MPK3, and MPK4/11 is unaffected in *rbohD* mutants, and flg22-induced oxidative burst is maintained in *mpk3 mpk6* mutants [36].

**Fig. 2:**
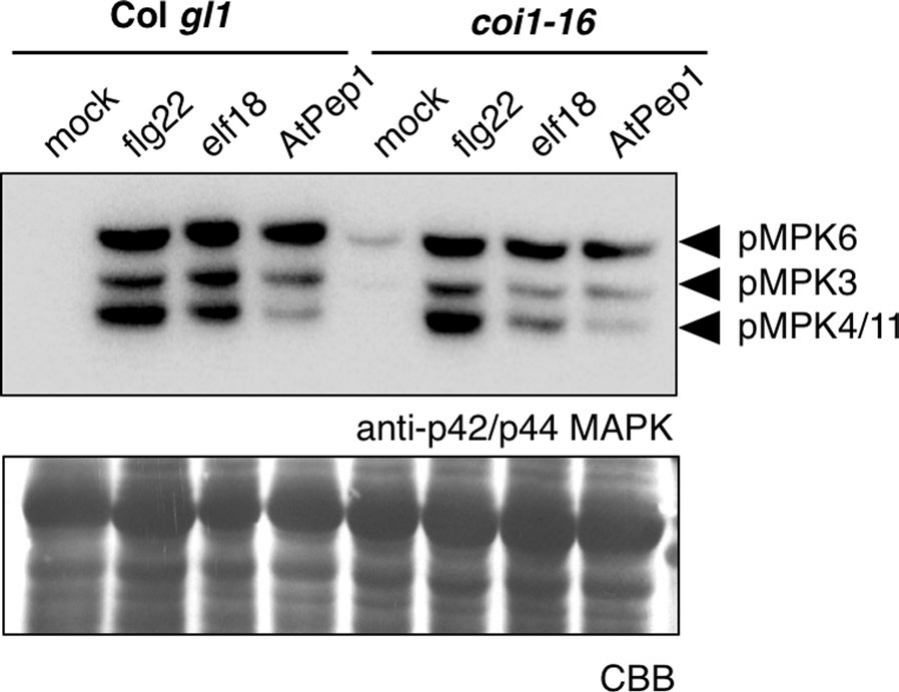
Elicitor-induced MAPK activation in *coi1*-*16* mutants. Phosphorylation of MPK6, MPK3, and MPK4/11 in 14-day-old seedlings treated with 200 nM flg22, 200 nM elf18, or 1 µM AtPep1 for 10 min compared to a mock control. Total protein was extracted from twelve seedlings and analyzed by immunoblot using anti-p42/p44 MAPK (Cell Signaling) and anti-rabbit-HRP (Sigma Aldrich) antibodies. The membrane was stained with Coomassie Brilliant Blue (CBB) as a measure of sample loading. Three experimental replicates were performed with similar results

We were thus interested to assess if other AtPep1-triggered responses, such as MAPK activation, were also genetically dependent on JA perception. To test this, we treated Col *gl1* and *coi1*-*16* plants with flg22, elf18, or AtPep1 for 10 min and compared the activation of MPK6, MPK3, and MPK4/11 using immunoblot analysis. While flg22-induced MAPK activation was comparable between *coi1*-*16* and control Col *gl1* plants, we observed slightly reduced MAPK activation in *coi1*-*16* mutants following treatment with elf18 and AtPep1 (Fig. 2). Although reduced, MAPKs were still activated by all three immunogenic elicitors in *coi1*-*16*, suggesting that JA perception is only partially required for AtPep1-induced MAPK activation.

**Fig. 3:**
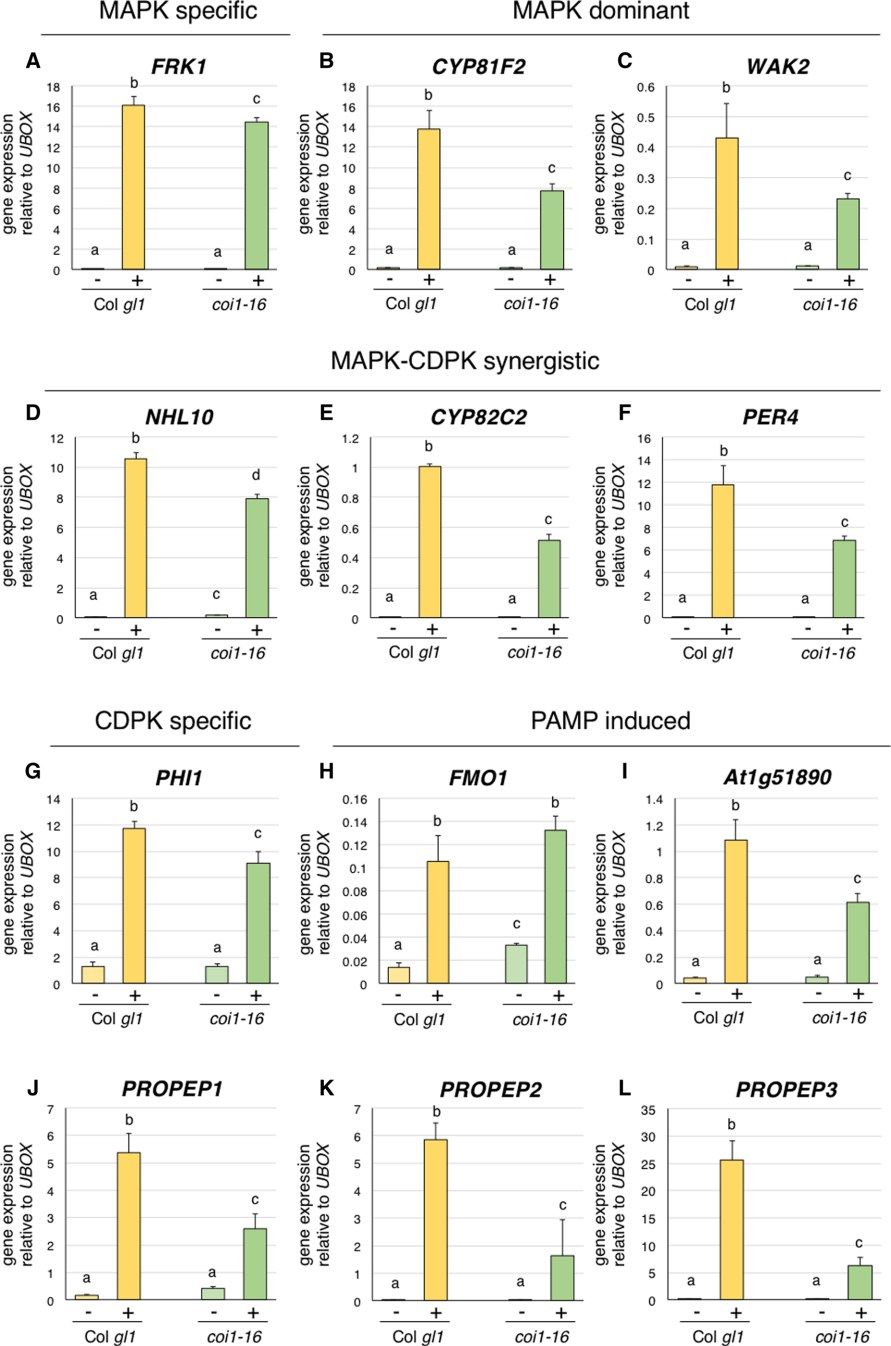
Analysis of AtPep1-induced gene expression in *coi1*-*16* mutants. Twelve 14-day-old Col *gl1* and *coi1*-*16* seedlings were treated with water (−) or 1 µM AtPep1 (+) for 120 min prior to RNA extraction. Quantitative real-time PCR was used to assess expression levels of the MAPK specific gene *FRK1* (**A**), the MAPK dominant genes *CYP81F2* (**B**) and *WAK2* (**C**), the MAPK-CDPK synergistic genes *NHL10* (**D**), *CYP82C2* (**E**), and *PER4* (**F**), the CDPK specific gene *PHI*-*1* (**G**), the PAMP-induced genes *FMO1* (**H**) and *At1g51890* (**i**), and the AtPep precursor genes *PROPEP1* (**J**)*, PROPEP2* (**K**), and *PROPEP3* (**L**). Values are means + standard deviations (*n *= 3 technical replicates from the same cDNA), normalized against the relative average expression of *UBOX* from the same sample. A total of three independent experimental replicates were performed with similar results. Statistically significant groups (*p* < 0.05) are indicated with lower-case letters based on a one-way ANOVA followed by Tukey’s post-test

Activated MAPKs are known to regulate transcriptional changes via phosphorylation of WRKY and other transcription factors [38], as are CDPKs [39, 40]. Transcript profiling experiments have delineated sets of genes that are dependent on MAPKs, CDPKs, or both, to varying levels [34]. Because we observed a slight reduction in MAPK activation in *coi1*-*16* mutants we were interested to test if MAPK-regulated gene expression was also affected. We found that although the MAPK-specific gene *FRK1* [34] and the MAPK-dominant genes *CYP81F2* and *WAK2* [34] were clearly induced in *coi1*-*16* mutants after AtPep1 treatment, they were expressed to significantly lower levels than in control Col *gl1* plants (Fig. 3A–C). A similar trend was observed when we compared AtPep1-induced expression of the MAPK-CDPK synergistic genes *NHL10, CYP82C2* and *PER4* [34] and the CDPK-specific gene *PHI*-*1* [34] (Fig. 3D–G). Induction of *At1g51890* [41] was also reduced in *coi1*-*16* mutants, however, interestingly, AtPep1-induced expression of *FMO1* [42] was similar in *coi1*-*16* and Col *gl1* (Fig. 3H–I).

AtPep1 is a 23-amino acid peptide processed from a precursor peptide encoded by *PROPEP1* [7]. *PROPEP1* is part of a six-member gene family in Arabidopsis [7], several members of which are induced by immune-related phytohormones such as MeSA and MeJA [8]. Treatment of Arabidopsis plants with AtPeps differentially induces expression of several precursor *PROPEPs* [8] and *PEPR1/2* [9], indicative of positive feedback that is often observed in signaling pathways. We found that AtPep1-induced expression of *PROPEP1, PROPEP2, PROPEP3,* and *PEPR1* was strongly reduced in *coi1*-*16* compared to Col *gl1* (Fig. 3J–L; Additional file 3), further supporting a role for JA perception in AtPep1-mediated signaling.

## Conclusions

Here we show that AtPep1-induced seedling growth inhibition and oxidative burst are strongly compromised in *coi1*-*16* mutants, which is in full agreement with results obtained in a previous study using the *coi1*-*1* allele [24]. We additionally show that AtPep1-induced MAPK activation and gene expression are maintained but reduced in *coi1*-*16* mutants. Together, our data suggest that JA perception via the COI1 receptor is involved in AtPep1-triggered responses. Future work is needed to decipher the mechanistic interplay between JA and AtPep signaling in the plant immune response.

## Limitations

- Immunological assays were conducted with saturating concentrations of eliciting peptides flg22, elf18, or AtPep1.
- Global transcript profiling was not conducted; only a panel of representative elicitor-induced genes was analyzed.

## Additional files

**Additional file 1: ** Primers used in this study. A list of primers used for qPCR.

**Additional file 2:** Fresh weight of seedlings grown in MS media. Fresh weight of seedlings 10 days after continual growth in sterile MS liquid media. Values are means + standard deviation (*n*=6 plants). Three biological replicates were performed with similar results. Statistically significant groups (*p* < 0.05) are indicated with lower-case letters based on a one-way ANOVA followed by Tukey’s post-test.

**Additional file 3: ** AtPep1-induced *PEPR1* expression in *coi1-16* mutants. Twelve 14-day-old Col *gl1* and *coi1-16* seedlings were treated with water (-) or 1 µM AtPep1 (+) for 120 minutes prior to RNA extraction. Quantitative real-time PCR was used to assess expression level of *PEPR1*. Values are means + standard deviations (*n*=3 technical replicates from the same cDNA), normalized against the relative average expression of *UBOX* from the same sample. Three independent biological replicates were performed with similar results. Statistically significant groups (*p* < 0.05) are indicated with lower-case letters based on a one-way ANOVA followed by Tukey’s post-test.

## Abbreviations

AOS: allene oxide synthase
AtPep: *Arabidopsis thaliana* peptide
BAK1: BRI1-associated receptor kinase 1
BIK1: botrytis-induced kinase 1
BRI1: brassinosteroid insenstive 1
Col-0: columbia-0
COI1: coronatine insensitive 1
CYP81F2: cytochrome P450, family 81, subfamily F, polypeptide 2
CYP82C2: cytochrome P450, family 82, subfamily C, polypeptide 2
DAMP: damage-associated molecular pattern
EFR: elongation factor-tu (EF-Tu) receptor
elf18: 18 amino acid peptide from bacterial EF-Tu
flg22: 22 amino acid peptide from bacterial flagellin
FLS2: flagellin sensitive 2
FMO1: flavin-dependent monoxygenase 1
FRK1: flg22-induced receptor-like kinase 1
GL1: glabra 1
JA: jasmonate
NHL10: NDR1/HIN1-like 10
MeJA: methyl-jasmonate
MeSA: methyl-salicylate
MPK3/4/6/11: mitogen-activated protein kinase 3/4/6/11
PAMP: pathogen-associated molecular pattern
PCR: polymerase chain reaction
PDF1.2: plant defensin 1.2
PEPR1/2: AtPep receptor 1/2
PER4: peroxidase 4
PHI-1: phosphate induced-1
PR-1: pathogenesis related-1
PROPEP1/2/3: AtPep precursor peptide1/2/3
PRR: pattern recognition receptor
ROS: reactive oxygen species
RBOHD: respiratory burst oxidase homologue D
RNA: ribonucleic acid
SA: salicylate
SID2: salycylic acid induction deficient 2
WAK2: wall-associated kinase 2
WRKY: WRKY-DNA binding protein
