# Supplementary material for: The jasmonate receptor COI1 is required for AtPep1-induced immune responses in Arabidopsis thaliana
Source: BMC Res Notes. 2018 Aug 3;11:555. doi: 10.1186/s13104-018-3628-7 (PMC6076402; doi:10.1186/s13104-018-3628-7)
Supplement: Supplementary file 1 — Additional file 1: Primers used in this study. A list of primers used for qPCR. [file 13104_2018_3628_MOESM1_ESM.pdf]

# Additional File 1: Primers used in this study.

| Primer name | Sequence (5’-3’) | Reference |
|---|---|---|
| qUBOX_F | TGCGCTGCCAGATAATACACTATT | [1] |
| qUBOX_R | TGCTGCCCAACATCAGGTT | |
| qFRK1_F | ATCTTCGCTTGGAGCTTCTC | [2] |
| qFRK1_R | TGCAGCGCAAGGACTAGAG | |
| qNHL10_F | TTCCTGTCCGTAACCCAAAC | [3] |
| qNHL10_R | CCCTCGTAGTAGGCATGAGC | |
| qFMO1_F | TGTGTTTGAAGATGGGACGACA | [4] |
| qFMO1_R | GTTCGAGCTGCTTTGGACGTAT | |
| qAt1g51890_F | CCAGTTTGTTCTGTAATACTCAGG | [2] |
| qAt1g51890_R | CTAGCCGACTTTGGGCTATC | |
| qCYP81F2_F | AATGGAGAGAGCAACACAATG | [2] |
| qCYP81F2_R | ATACTGAGCATGAGCCCTTTG | |
| qWAK2_F | CGTGTGAGTACACAAATCATCG | [3] |
| qWAK2_R | TGGTTTAACCTCCTTTGTCTTC | |
| qCYP82C2_F | AATCTACCTGCCTGGCACTG | [3] |
| qCYP82C2_R | GAGAAATGGCCCATGTAAGG | |
| qPER4_F | CGTTTAGGGCTATCGCAGAC | [3] |
| qPER4_R | ACGTGAGGCATTGAGCTTG | |
| qPHI1_F | TTGGTTTAGACGGGATGGTG | [3] |
| qPHI1_R | ACTCCAGTACAAGCCGATCC | |
| qPEPR1_F | CGCTTTCAAAACCGTGAGGG | This study |
| qPEPR1_R | CCGCTCTCTTCCTCGTAACC | |
| qPROPEP1_F | CAAGATTCTCCGACAACGTC | This study |
| qPROPEP1_R | GTCACAACGACCTCCTCCTT | |
| qPROPEP2_F | CGACCAAGCTCTCATAGCT | This study |
| qPROPEP2_R | GGACGACCTGAACTAGGCTT | |
| qPROPEP3_F | GCGAGGAAGATGAGAGTATCGA | This study |
| qPROPEP3_R | GGTCATGCCATCTTCTTCTT | |

# References


1. Nemhauser JL, Mockler TC, Chory J. Interdependency of Brassinosteroid and Auxin Signaling in Arabidopsis. PLoS Biol.2004;2:e258.

2. He P, Shan L, Lin NC, Martin GB, Kemmerling B, Nurnberger T, et al. Specific bacterial suppressors of MAMP signaling upstream of MAPKKK in Arabidopsis innate immunity.

Cell. 2006;125:563–75.

3. Boudsocq M, Willmann MR, McCormack M, Lee H, Shan L, He P, et al. Differential innate immune signalling via Ca2+ sensor protein kinases. Nature. 2010;464:418–22.

4. Sohn KH, Segonzac C, Rallapalli G, Sarris PF, Woo JY, Williams SJ, et al. The Nuclear Immune Receptor RPS4 Is Required for RRS1$^{SLH1}$-Dependent Constitutive Defense Activation in Arabidopsis thaliana. PLoS Genet. 2014;10:e1004655.
