# Supplementary material for: The jasmonate receptor COI1 is required for AtPep1-induced immune responses in Arabidopsis thaliana
Source: BMC Res Notes. 2018 Aug 3;11:555. doi: 10.1186/s13104-018-3628-7 (PMC6076402; doi:10.1186/s13104-018-3628-7)
Supplement: Supplementary file 2 — Additional file 2: Fresh weight of seedlings grown in MS media. Fresh weight of seedlings 10 days after continual growth in sterile MS liquid media. Values are means + standard deviation (n=6 plants). Three biological replicates were performed with similar results. Statistically significant groups (p < 0.05) are indicated with lower-case letters based on a one-way ANOVA followed by Tukey’s post-test. [file 13104_2018_3628_MOESM2_ESM.pdf]

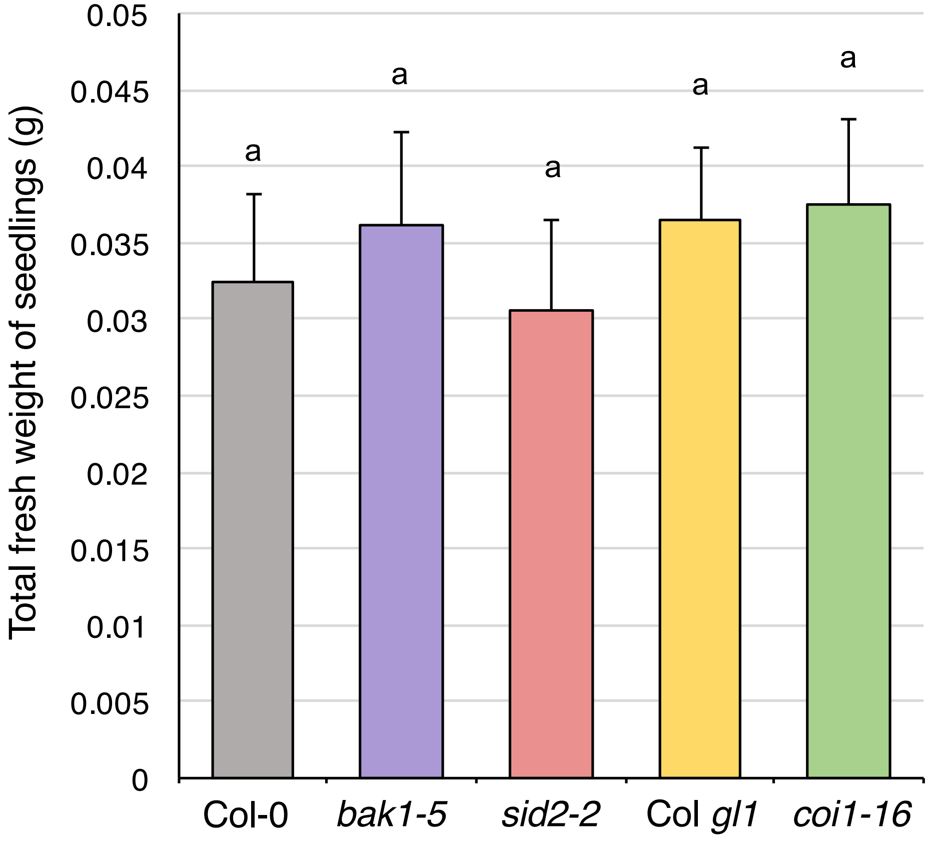

Additional File 2: Fresh weight of seedlings grown in MS media.

Fresh weight of seedlings 10 days after continual growth in sterile MS liquid media. Values are means + standard deviation (n=6 plants). Three biological replicates were performed with similar results. Statistically significant groups ($p < 0.05$) are indicated with lower-case letters based on a one-way ANOVA followed by Tukey's post-test.
