# Supplementary material for: The jasmonate receptor COI1 is required for AtPep1-induced immune responses in Arabidopsis thaliana
Source: BMC Res Notes. 2018 Aug 3;11:555. doi: 10.1186/s13104-018-3628-7 (PMC6076402; doi:10.1186/s13104-018-3628-7)
Supplement: Supplementary file 3 — Additional file 3: AtPep1-induced PEPR1 expression in coi1-16 mutants. Twelve 14-day-old Col gl1 and coi1-16 seedlings were treated with water (-) or 1 µM AtPep1 (+) for 120 minutes prior to RNA extraction. Quantitative real-time PCR was used to assess expression level of PEPR1. Values are means + standard deviations (n=3 technical replicates from the same cDNA), normalized against the relative average expression of UBOX from the same sample. Three independent biological replicates were performed with similar results. Statistically significant groups (p < 0.05) are indicated with lower-case letters based on a one-way ANOVA followed by Tukey’s post-test. [file 13104_2018_3628_MOESM3_ESM.pdf]

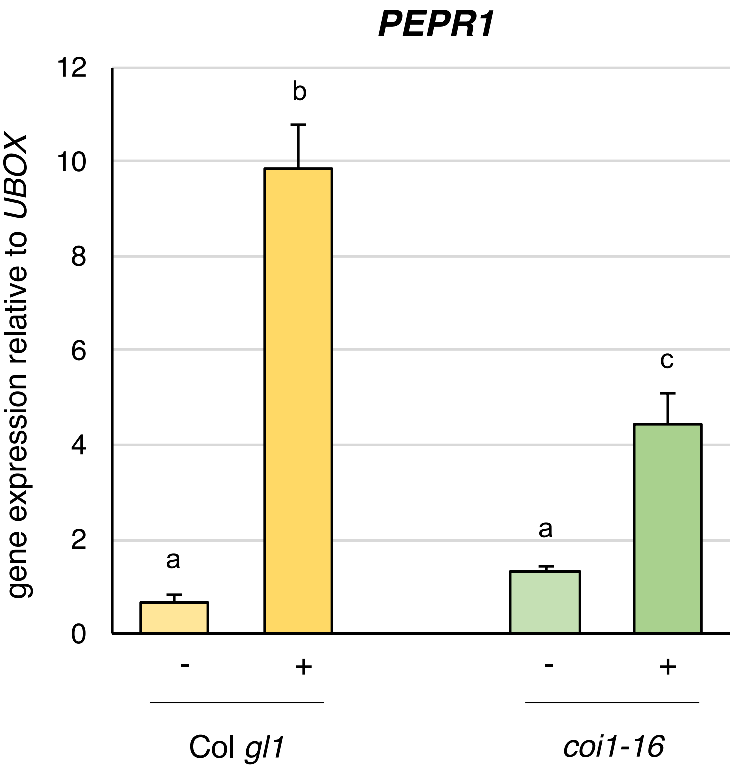

Additional File 3: AtPep1-induced *PEPR1* expression in *coi1-16* mutants.

Twelve 14-day-old Col *gl1* and *coi1-16* seedlings were treated with water (-) or 1 μM AtPep1 (+) for 120 minutes prior to RNA extraction. Quantitative real-time PCR was used to assess expression level of *PEPR1*. Values are means + standard deviations (n=3 technical replicates from the same cDNA), normalized against the relative average expression of *UBOX* from the same sample. A total of three independent biological replicates were performed with similar results. Statistically significant groups ($p < 0.05$) are indicated with lower-case letters based on a one-way ANOVA followed by Tukey's post-test.
